# Synthesis of Mesoporous Silica Sol with Low Refractive Properties for Increasing Transmittance

**DOI:** 10.3390/mi15070892

**Published:** 2024-07-08

**Authors:** Han-San Ko, Misun Kang, Jong-tak Lee, Jae Young Bae

**Affiliations:** Department of Chemistry, Keimyung University, Daegu 42601, Republic of Korea; hansanss@naver.com (H.-S.K.); misun.kang@gmail.com (M.K.); atomos27@daum.net (J.-t.L.)

**Keywords:** anti-reflective coating, single-layer coating, mesoporous silica, low refractive index

## Abstract

Currently, coating with anti-reflective materials is an attractive approach to improve the quality of screen-based displays. In this study, mesoporous silica particles were systematically synthesized as a function of surfactant (i.e., CTAC-cetyltrimethylammonium chloride) concentration to serve as main coating fillers possessing low refractive indices. Precisely changing the amount of the CTAC surfactant, silica sol with an average diameter of 50 nm exhibits distinctively different specific surface areas, pore size, and pore volume. Prior to the preparation of final coating solutions containing these silica particle fillers, the percentage of solid content was optimized on a glass slide. The use of 50 wt% solid content exhibited the highest transmittance of light. Among various content levels of silica sol, the use of 3.5 wt% of silica particles in the solid content displayed the highest transmittance (i.e., best anti-reflectiveness). Under the almost identical coating layers prepared with the fixed amount of silica particles possessing different surface areas, pore size, and pore volume, it appears that the largest pore volume played the most important role in improving the anti-reflective properties. Experimentally understanding the key feature of low-refractive filler materials under the optimized conditions could provide a clear view to develop highly effective anti-reflective materials for various display applications.

## 1. Introduction

Anti-reflective (AR) coating plays an important role in improving device performance, boosting energy efficiency, and increasing visual comfort [1,2,3,4]. These coatings, when applied to glass surfaces, increase transmittance by minimizing light reflection, making them valuable for various applications such as displays, solar cells, and smart windows [5,6,7]. Anti-reflection performance can be achieved through two primary methods. The first method involves the use of the moth-eye technique [8,9,10,11], which generates surface irregularities at the microscopic level. These irregularities scatter incoming light, significantly reducing reflectivity through diffuse reflection effects. However, as touch screen applications, the moth-eye method encounters significant challenges due to its inherent characteristics. One major issue is the difficulty in removing surface contaminants, which can accumulate over time and degrade performance. Furthermore, the method exhibits poor durability under frequent use, as the delicate structures are prone to wear and tear. This compromises the long-term effectiveness of the anti-reflective properties, making it less suitable for touch screen applications where consistent interaction is expected. The second method is to increase the transmittance of the glass to reduce light re-flection [12,13,14,15]. Unlike the moth-eye technique, this method does not scatter light but instead enhances the glass’s inherent transparency. Currently, AR coatings that enhance transmittance are widely utilized in smart displays globally. One prevalent technique for increasing transmittance is Physical Vapor Deposition (PVD) [16,17,18]. This method enhances transmittance by alternately stacking materials with low and high refractive in-dices. The PVD method achieves excellent quality by finely adjusting the thickness of the deposition sample [19,20], allowing precise control of the refractive index. However, vacuum deposition, a key aspect of PVD, is costly, significantly increasing the price of cover glass materials. To address these drawbacks, research has been actively conducted on wet coating at atmospheric pressure as an alternative to vacuum deposition for several years. Similar to vacuum deposition, wet coating also involves a multi-coating method where materials with low and high refractive indices are alternately applied [21,22,23]. However, this method has disadvantages, such as difficulty in adjusting the thickness and the requirement for multiple processing steps to protect the previous surface [24,25,26,27]. Recently, studies have mainly focused on increasing the transmittance of a cover glass by using ultra-low refractive index materials in a single-layer coating [28,29,30]. This method enhances light transmittance with the advantage of a thinner coating.

The most important aspect of the ultra-low refractive single-layer coating method is the ultra-low refractive index material used for coating, with hollow silica being the most popular choice [31,32,33,34]. Hollow silica particles have a very low refractive index because they contain a significant amount of air, which has the lowest theoretical refractive index due to its internal hollow structure. In addition, to maximize the transmittance increase, the particle size should be less than 100 nm. However, synthesizing hollow silica with a uniform size of 100 nm or less is challenging [35,36,37]. Even if uniformity is achieved, synthesizing it in high concentrations is difficult, hindering its application in smart displays. To address the limitations, research on AR coatings using mesoporous silica is currently active [38,39,40,41]. Mesoporous silica, with its higher theoretical refractive index due to lower pore volume compared to hollow silica, is more suitable for smart displays. It is easier to synthesize and allows for better control of particle size.

This study proposes a method for synthesizing a mesoporous silica sol that may realize an effective anti-reflective coating without going through a heat treatment process. This approach aims to increase the transmittance of glass while simplifying the manufacturing process and reducing costs. To synthesize a mesoporous silica sol with various pore volumes, different mesoporous silica sols were synthesized by adjusting the amount of surfactants. The surfactant was removed using a removal extraction method without heat treatment. Comparing the properties of the obtained mesoporous silica sol, it was confirmed that the pore volume and the specific surface area varied depending on the amount of the surfactants. In this study, to enhance the transmittance of glass by coating it with mesoporous silica sol, we aim to analyze which factor, pore volume, or specific surface area, plays a more crucial role. Through this analysis, we intend to reveal the advantages that mesoporous silica coating provides as an anti-reflective coating and to determine the possibility of maximizing the efficiency of the anti-reflective coating.

## 2. Materials and Methods

### 2.1. Reagents

The chemicals used in this study were cetyltrimethylammonium chloride (CTAC, 25 wt%, Aldrich, Saint Louis, MO, USA), tetraethylorthosilicate (TEOS, 98%, Acros, Loughborough, UK), glycidoxypropyltrimethoxysilane (GPTMS, 95%, Daejung, Cheongju, Republic of Korea), methyltriethoxysilane (MTES, 97%, Acros, Loughborough, UK), triethanolamine (TEA, 99%, Aldrich, Darmstadt, Germany), hydrochloric acid (HCl, 36%, Samchun, Pyeongtaek, Republic of Korea), ethanol (EtOH, 99.5%, Duksan, Ansan, Republic of Korea), and iso-propyl alcohol (99.5%, Duksan, Ansan, Republic of Korea). These were used without any purification.

#### 2.1.1. Mechanism of the Synthesis Process of Mesoporous Silica

**Figure 1:**
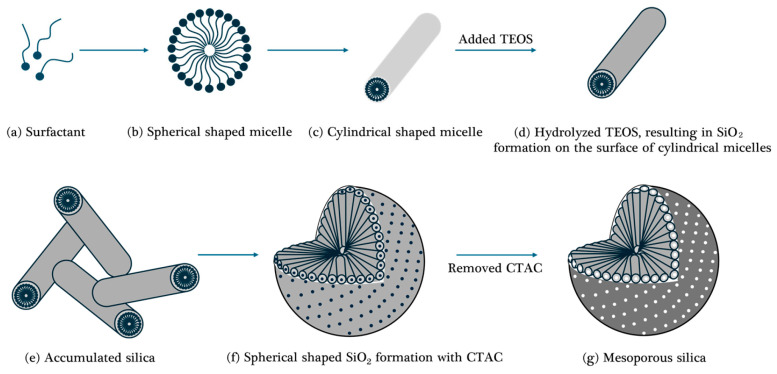
Synthetic process of mesoporous silica sol in the presence of CTAC surfactant.

In this study, a cationic surfactant called CTAC was used as a template, and TEOS was used as a silica precursor. Mesoporous silica was prepared using a surfactant, as illustrated in Figure 1. Initially, when the concentration of the surfactant reaches a specific threshold known as the critical micelle concentration (CMC)-1, spherical micelles are formed (shown in Figure 1b). Upon further increase in surfactant concentration to CMC-2, the spherical micelles grow into cylindrical shapes (shown in Figure 1c). Simultaneously, hydrolyzed TEOS, bearing a negative charge, gathers near the positively charged amine groups on the surface of cylindrical micelles, leading to the formation of SiO_2_ (shown in Figure 1d). These SiO_2_-formed micelles then begin to accumulate (shown in Figure 1e). Through these reactions, the silica precursor is converted into SiO_2_, and the SiO_2_ formed cylindrical micelles aggregate together to form a spherical shape (shown in Figure 1f). The spherical-shaped micelles with silica were generated in a cylindrical shape due to the internal surfactant, and then the surfactant was removed via an extraction method to form spherical silica with mesoporous structure (shown in Figure 1g) [42]. In the synthesis of silica, a basic catalyst is required to induce the condensation reaction, and the most commonly used basic catalyst is an amine group. However, in general, the presence of basic amine groups increases the rate of the condensation reaction, thereby acting as a decisive factor in increasing the size of the silica particles. Therefore, a weak base (TEA) with a relatively low pH among organic amines was selected to reduce the reaction rate and consequently produce smaller mesoporous silica particles.

#### 2.1.2. Synthesis Process of Mesoporous Silica Sol

After adding a certain amount of CTAC to water as a solvent, TEA was added to adjust the pH to a basic level. Following that, the mixture was stirred at 60 °C for 5 min to ensure homogeneity. Then, the silica precursor, TEOS, was added and stirred at 60 °C for 2 h. Following the stirring process, the pH was neutralized with hydrochloric acid, resulting in the formation of a white slurry, which was separated through centrifugation. The obtained slurry was dispersed in a solution containing 1.0 M HCl/EtOH and stirred for one day to remove the surfactant. After the surfactant removal process, the white slurry was separated using a centrifuge, and was then dispersed in EtOH with a solid content of 20 wt% to prepare it in sol form. The sample was named MSS (Mesoporous Silica Sol).

### 2.2. Synthesis of Silane Coating Solution for Glass Fabric

The synthesized mesoporous silica sol was utilized to formulate the coating solution, where the AR coating primarily consisted of alkoxy silanes as the main inorganic component. Specifically, GPTMS, MTES, and TEOS were dispersed in an IPA solvent in a ratio of 5:3:2. To prepare the coating solution, water and hydrochloric acid were added to adjust the pH to 3, and the mixture was stirred at room temperature for 3 h.

### 2.3. Analytical Instruments and Conditions

Transmission electron microscope (FE-TEM; HF-3300, Hitachi High-Technologies Corporation, Tokyo, Japan) analysis was conducted to analyze the pore size and particle size of mesoporous silica. The samples were dispersed in ethanol, and a small amount was collected on a Cu grid, dried, and analyzed at an accelerated voltage of 300 kV.

Scanning electron microscope (SEM; JSM-IT500, JEOL, Tokyo, Japan) analysis was conducted to analyze the thickness of the coating layer. The samples were coated with platinum and analyzed at an accelerated voltage of 10 kV.

The pore structure of the mesoporous silica was analyzed using X-ray diffraction (XRD; X’pert PRO MPD, PANalytical, Malvern, UK). The measurements were conducted in 2θ scan mode. Cu-Ka radiation (λ = 0.154 nm) was employed as the X-ray sources. The mesoporous hollow silica spheres typically do not exhibit X-ray scattering peaks due to their irregular atomic arrangement. However, when the pores are arranged regularly, a diffraction peak appears at a low angle, commonly at 2θ = 10° or less, indicating the regularity of the pore arrangement. Analysis of these peaks provides insights into the shape, arrangement, and size of pores.

N_2_-sorption (Quadrasorb Si, QUANTACHROME, Boynton Beach, FL, USA) was employed to determine the specific surface area and pore distribution of mesoporous hollow silica. The measured temperature was maintained at 77 K using liquid nitrogen, and the adsorbed nitrogen was normalized to standard temperature and pressure. Before sample analysis, the sample was annealed at 200 °C for approximately 2 h to remove moisture and residual organic matter adsorbed on the surface.

UV-Vis spectroscopy (Agilent Technologies Cary 60, Agilent, Santa Clara, CA, USA) was used to analyze the transmittance of the coated substrate. The device operated in transmittance mode within the wavelength range of 400 to 700 nm. Initially, the blank was measured in its bare state, and, subsequently, the transmittance was measured after applying the coating material to the substrate.

## 3. Results and Discussion

### 3.1. Mesoprous Silica

#### 3.1.1. Analysis of TEM

Mesoporous silica was synthesized by varying the amount of a surfactant, CTAC, as shown in Table 1.

**Figure 2:**
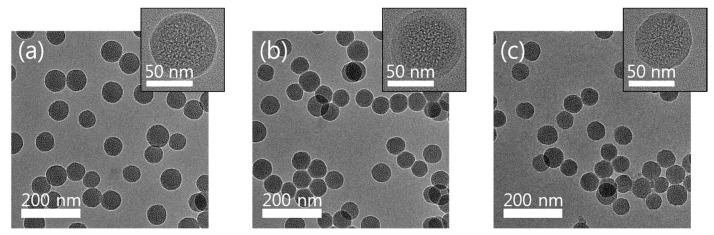
TEM image of (**a**) MSS-1, (**b**) MSS-2, and (**c**) MSS-3.

When the molar ratio of TEOS to CTAC was 44.6:1, the particle size was too large (not presented in this paper). Conversely, when the molar ratio was 11.2:1, proper formation of mesoporous structure did not occur. As shown in Figure 2, the particles’ size for all three samples was about 50 nm, and the pores could be observed in the TEM images.

#### 3.1.2. Analysis of XRD

**Figure 3:**
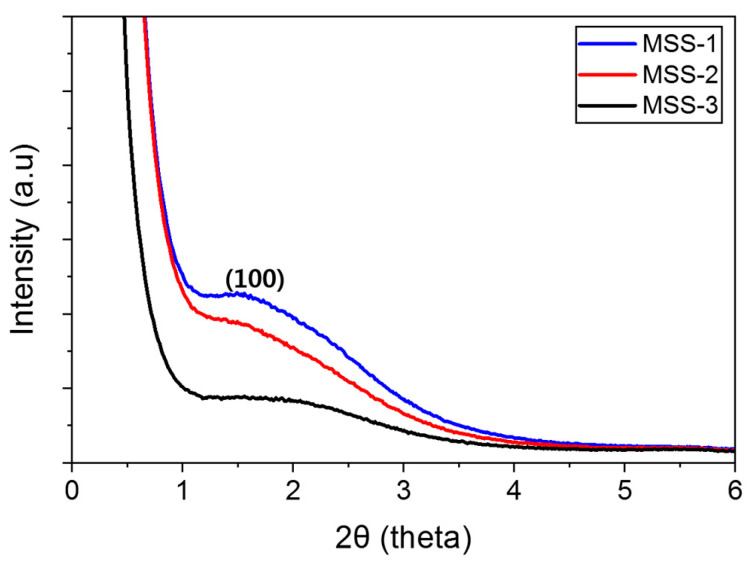
XRD diffractograms of three mesoporous silica particles.

The synthesized mesoporous silica was obtained in powder form, and XRD analysis was performed. The results are depicted in Figure 3.

It was confirmed that MSS-1 was relatively well formed when the intensity values were higher and relatively sharper than other samples at 2θ around 1.5 degrees. Powder X-ray diffraction pattern indicates the crystallinity of materials. As our silica particles are highly amorphous, the distinctively broad peaks at low angle diffraction (i.e., 2θ angle in the range of 1.6–2.2° for (100) plane) suggested the presence of long-range ordering of the pores in the silica nanoparticles (28). Although these particles are not highly ordered hexagonal structures due to absence of (110) and (200) planes over 2θ of 4°, our silica particles with detectably broad peaks exhibited somewhat less ordered mesoporous features, which are good enough for testing in the anti-reflective coating application [43,44].

#### 3.1.3. Analysis of N_2_-Sorption

**Figure 4:**
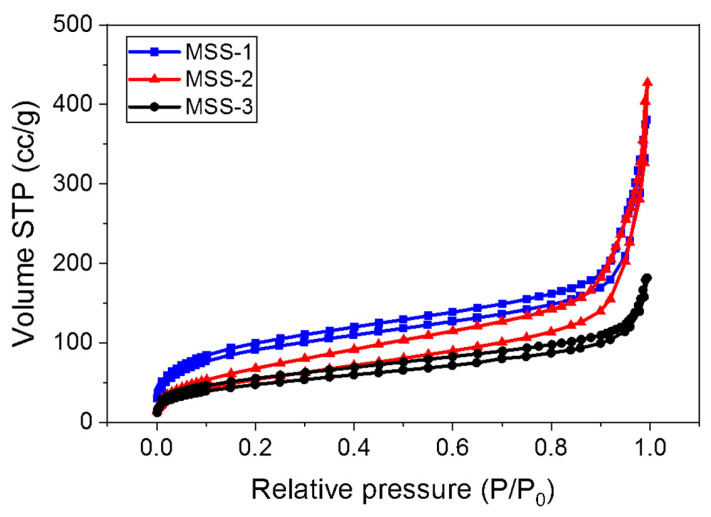
BET isotherms of three different mesoporous silica using N_2_ gas.

The synthesized mesoporous silica was obtained in powder form, and N_2_-sorption analysis was conducted. The results are shown in Figure 4.

In Figure 4, the presence of pores was confirmed by the splitting of the adsorption and desorption peaks around a relative pressure of 0.1~0.2. Additionally, the extension of the adsorption peak to a relative pressure of 0.3 indicates that MSS-1 exhibited the highest volume value and the best specific surface area. On the other hand, MSS-2 showed the widest area between the split adsorption and desorption peaks, indicating the highest final adsorption point and, therefore, the highest pore volume value. Based on these results, the BJH pore size distribution was measured, and the results are presented in Table 2.

In BJH results, although the average pore size value of MSS-2 was the smallest compared to the other materials, the area under the adsorption and desorption curve of MSS-2 was larger, indicating that MSS-2 had the highest pore volume. This can be confirmed by examining MSS-1, which shows a small pore volume due to insufficient pore formation when the amount of surfactant is limited. In the case of MSS-2, the pore structure is well formed with an appropriate amount of surfactant, resulting in a large pore volume. Conversely, for MSS-3, the excessive amount of surfactant leads to the collapse of the pore structure, causing clogging and consequently a decrease in pore volume. Therefore, for further experiments, a coating solution was prepared using these mesoporous silica sols applied to a glass substrate and subjected to transmittance analysis.

### 3.2. Transmittance Analysis

#### 3.2.1. Setting of Coating Solution for High Transmittance

An experiment was conducted to determine the appropriate content of the coating solution for application on the glass substrate. Each experiment was performed with varying contents of solids (GPTMS + MTES + TEOS) and silica sol, and the results are presented in Table 3 and Table 4, respectively. The coating was applied using the flow coating method, followed by curing at 120 °C for 30 min. The SEM images revealed that our coating process produced layers with nearly identical thickness, regardless of the solid content in the coating solution. Thus, the transmittance of the film was primarily influenced by the amount of solid content rather than the thickness of the coated layer.

The average transmittance of bare glass was 90.0%. The highest transmittance was achieved with coatings containing 50 wt% concentration. Consequently, all subsequent transmittance experiments were conducted by adding silica based on a 50 wt% ratio.

**Figure 5:**
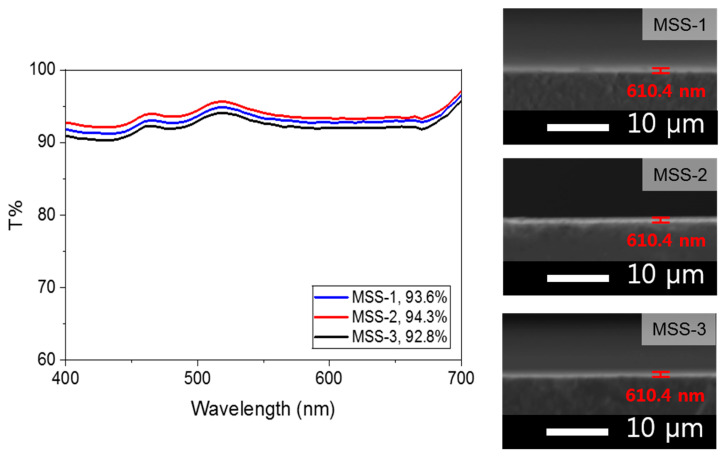
Transmittance result of various MSS types and corresponding SEM images.

As indicated in Table 4, the highest transmittance of 93.6% was achieved when the silica content was 3.5 wt% relative to the weight of the coating solution, with a solid content concentration of 50 wt%. In addition, at higher contents of mesoporous silica, some areas appeared partially hazy when observed with the naked eye. Therefore, after adjusting the silica content to 3.5% based on the weight of the coating solution, the transmittance of each type of the mesoporous silica was compared. The results are shown in Figure 5.

#### 3.2.2. Transmittance Comparison of MSS Types

As depicted in Figure 5, it was confirmed that the highest transmittance values were measured at 525 nm. This trend is similar to the result of V-coat style outcomes, where a low-refractive-index sample is coated in a single layer to increase transmittance. Thus, increasing transmittance using these three samples appears to be successful, with MSS-2 exhibiting the highest transmittance of 94.3%. This suggests that the transmittance is more closely related to pore volume than to specific surface area. This implied that the air, having the lowest refractive index, plays a critical role. It is important to note that the thickness of the layer remained consistent, given the equal silica particle content (i.e., 3.5 wt%) in the coating solution. Thus, it can be inferred that the composite layer containing silica particles with the largest internal pore volume exhibited the highest transmittance. We hypothesize that the increased pore volume significantly enhances the anti-reflective properties by trapping more air, thereby achieving the lowest refractive index.

## 4. Conclusions

In this study, mesoporous silica sol and coating solution was prepared for application as an anti-reflective coating on the surface of smart displays. This was achieved through surfactant-assisted synthesis using TEOS as a precursor. It was easily synthesized cost effectively through the removal extraction method without the need for heat treatment. We found that optimizing the surfactant quantity during the synthesis of the mesoporous silica sol, with a molar ratio of TEOS to CTAC at 25.5:1 (MSS-1), yielded the highest specific surface area. Furthermore, the molar ratio of TEOS to CTAC at 17.9:1 in MSS-2 indicated that the material had the largest pore volume. To evaluate the efficiency of the anti-reflective coating, the synthesized mesoporous silica sol was incorporated into the silane coating solution, applied onto a glass substrate, and the transmittance was measured. For the experiments, the preparation conditions were set at 50 wt% for solid content and 3.5 wt% for the silica content for comparison. Consequently, the highest transmittance was observed in the coating solution using MSS-2. This suggests that pore volume has a more significant impact on determining transmittance than the specific surface area of the material. By incorporating the synthesized mesoporous silica sol into a coating solution, we demonstrated that high transmittance can be achieved cost effectively for application, such as with displays and solar cells. Furthermore, the synthesized mesoporous silica highlights the importance of pore volume not only for smart displays and solar cells, but also for the adsorption of harmful substances and gases in the environmental field, addressing current issues and showcasing its potential for use in this area.

## Figures and Tables

**Table 1:** Synthesis table of mesoporous silica particles.

Sample	TEOS (mmol)	CTAC (mmol)	Molar Ratio (TEOS:CTAC)	D.I. Water (mL)
MSS-1	35.7	1.4	25.5:1	45
MSS-2	35.7	2.0	17.9:1	45
MSS-3	35.7	2.6	13.7:1	45

**Table 2:** SBET, pore size, and pore volume of three mesoporous silica particles.

Sample	Specific Surface Area (m^2^/g)	Pore Size (nm)	Pore Volume (cc/g)
MSS-1	338.3	3.4	0.465
MSS-2	205.5	3.0	0.610
MSS-3	177.7	3.4	0.218

**Table 3:** Effect of solid content for coating solution and corresponding SEM images (side view).

Solid Content	Bare Glass	30 wt%	50 wt%	70 wt%
Transmittance (T%)	90.0 ± 0.01	91.0 ± 0.03	91.1 ± 0.02	90.4 ± 0.02
	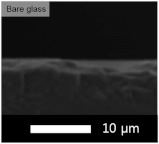	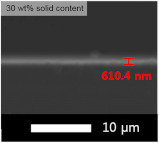	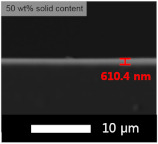	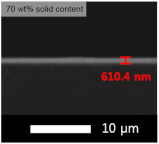

**Table 4:** Effect of silica particle content for coating solution and corresponding digital photos (top view) as well as their SEM images (side view).

Silica Sol Content	2.5 wt%	3.5 wt%	5.0 wt%	10 wt%
Transmittance (T%)	92.1 ± 0.02	93.6 ± 0.03	92.5 ± 0.03	91.6 ± 0.02
	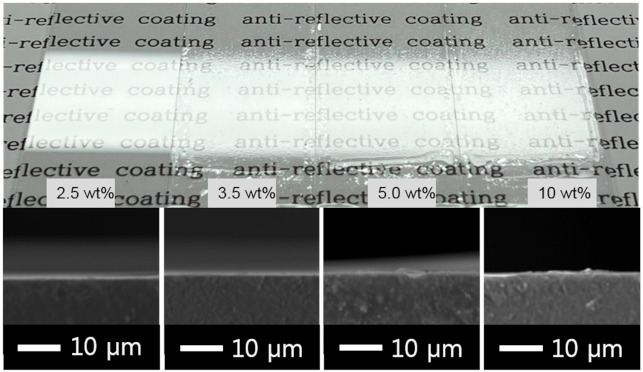			

## Data Availability

The original contributions presented in the study are included in the article, further inquiries can be directed to the corresponding author.
